# Determination of the Aspect-ratio Distribution of Gold Nanorods in a Colloidal Solution using UV-visible absorption spectroscopy

**DOI:** 10.1038/s41598-019-53621-4

**Published:** 2019-11-25

**Authors:** Rahul Kumar, Leonardo Binetti, T. Hien Nguyen, Lourdes S. M. Alwis, Arti Agrawal, Tong Sun, Kenneth T. V. Grattan

**Affiliations:** 1 School of Mathematics, Computer Science and Engineering, City University of London, London, EC1V 0HB United Kingdom; 2 School of Engineering and The Built Environment, Edinburgh Napier University, Edinburgh, EH10 5DT United Kingdom; 3 Department of Engineering and Information Technology, University of Technology Sydney, 15 Broadway, Ultimo, NSW 2007 Australia

**Keywords:** Characterization and analytical techniques, Optical spectroscopy

## Abstract

Knowledge of the distribution of the aspect ratios (ARs) in a chemically-synthesized colloidal solution of Gold Nano Rods (GNRs) is an important measure in determining the quality of synthesis, and consequently the performance of the GNRs generated for various applications. In this work, an algorithm has been developed based on the Bellman Principle of Optimality to readily determine the AR distribution of synthesized GNRs in colloidal solutions. This is achieved by theoretically fitting the longitudinal plasmon resonance of GNRs obtained by UV-visible spectroscopy. The AR distribution obtained from the use of the algorithm developed have shown good agreement with those theoretically generated one as well as with the previously reported results. After bench-marking, the algorithm has been applied to determine the mean and standard deviation of the AR distribution of two GNRs solutions synthesized and examined in this work. The comparison with experimentally derived results from the use of expensive Transmission Electron Microscopic images and Dynamic Light Scattering technique shows that the algorithm developed offers a fast and thus potentially cost-effective solution to determine the quality of the synthesized GNRs specifically needed for many potential applications for the advanced sensor systems.

## Introduction

Gold Nano Rods (GNRs), due to their asymmetric structure and unlike other symmetrical Gold Nanoparticles (GNPs) such as spheres, shells, cages and cubes exhibit two plasmonic resonances^1^. The plasmonic resonance corresponding to the shorter wavelength (around 520 nm), known as the Transverse Resonance (TR), arises due to the collective oscillation of free electrons in the presence of an external electromagnetic field along the transverse dimension of the GNRs whereas, the resonance at the longer wavelength, known as Longitudinal Resonance (LR), is due to the oscillation of free electrons along the longitudinal dimension. Due to the dependence of the position of the LR on their Aspect Ratio (the length (L) divided by the width (W) and designated here by AR) and the external refractive index, supported by well understood surface chemistry^2–5^, GNRs have found applications in a wide variety of fields which are as diverse as biomedical sciences^6–9^, sensor development^10–12^, imaging^13,14^ and electronics-based applications such as LEDs^15^ and solar cells^16^ development.

‘Bottom-up’ synthesis methods such as the seeded method^17^, the electrochemical reduction method^18^ or photochemical reduction^19^ of GNRs generally produce poly-dispersed solutions with various ARs of the GNRs. Therefore, knowing the distribution of the ARs in the synthesized colloidal solution is important as it reveals the quality of the solution and hence its potential to be used in many possible applications. For example, it is important to have a narrow size distribution in solution for uniform and long self-assemblies of GNRs^20^. In this regard, Transmission Electron Microscopy (TEM) provides a very effective means for determining the actual size of the GNRs but obtaining a statistically accurate size distribution of the colloidal solution is difficult because of the limited numbers of GNRs spanning over a few selected images which are used to calculate the average AR. Further, GNRs tend to ‘self-sort’ based on their sizes and shapes while drying on the TEM grid and therefore the selection of the position for imaging become important^21^. The measurement of even hundreds of GNRs from TEM images is an onerous process and most importantly, TEM devices are not readily accessible to all research groups. In recent years, use of Dynamic Light Scattering (DLS) techniques have been introduced to determine the size distribution of nanoparticles but this approach is limited to spherical particles and it cannot be used for anisotropic particles, such as GNRs.

In previous work, Eustis & Sayed^22^ have demonstrated that Mie-Gans theory could be used to determine the distribution of ARs in a given solution, by theoretically fitting the inhomogeneously-broadened LR absorption spectrum obtained using UV-Visible spectrometer. However, the fitting technique used was manual and therefore very cumbersome. Further, all the fittings employed were based on the assumption that the solvent used was pure deionized water and therefore the effect of contamination or impurities in the solvent were ignored. In this work, these important drawbacks are addressed by developing an algorithm based on the Bellman Principle of Optimality, to automate the curve-fitting process. The effect of the external medium was considered by taking into account the dielectric constant, ${\epsilon }_{m}$, of the solvent, while the fitting process was undertaken.

## Background theory and Algorithm formulation

According to the modified Gans Model^23^, the absorption coefficient ($\gamma$) of the GNRs in the colloidal solution considered is given by Eq. 1.

1 $$\gamma =\frac{2\pi NV{\epsilon }_{m}^{\frac{3}{2}}}{3\lambda }\sum _{J=A,B,C}\frac{(1/{P}_{J}^{2}){\epsilon }_{2}}{{\left({\epsilon }_{1}+\left[\frac{1-{P}_{J}}{{P}_{J}}\right]{\epsilon }_{m}\right)}^{2}+{\epsilon }_{2}^{2}},$$

where,

2 $${P}_{A}=\frac{1-{e}^{2}}{{e}^{2}}\left[\frac{1}{2e}ln\left(\frac{1+e}{1-e}\right)-1\right],$$

3 $${P}_{B}={P}_{C}=\frac{1-{P}_{A}}{2},$$

and

4 $$e=\sqrt{1-{\left(\frac{W}{L}\right)}^{2}}.$$

In the above equations, ${P}_{A}$, ${P}_{B}$ and ${P}_{C}$ are dependent on the shape of the GNRs as they also depend on the variable, $e$, which in turn is an inverse function of the AR, i.e. the ratio of the length (L) to width (W). N is number of GNRs per unit volume, V is the volume per particle, $\lambda$ is the wavelength, ${\epsilon }_{m}$ is the dielectric constant of the surrounding medium and ${\epsilon }_{1}$ and ${\epsilon }_{2}$ represent respectively the real and imaginary parts of the permittivity for the gold used.

**Figure 1:**
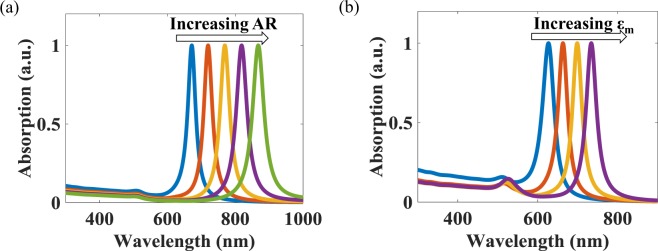
Self-normalized modelled absorption spectra showing the red shift in the Longitudinal Plasmon Resonance due to an increase in (**a**) the Aspect Ratio (AR) and (**b**) the Permittivity of the surrounding medium (${\epsilon }_{m}$).

In this work, the permittivity of the gold used was calculated by interpolating the data given by Johnson & Cristy^24^. Using an approach that is similar to that seen in the previous work, as reported by Eustis & Sayed. in^22^, V is considered as a constant, as, the average volume of the GNRs remains constant across the ARs in most of the seed synthesis methods. Figure 1(a,b) show the modelled dependence of the AR and ${\epsilon }_{m}$ respectively on the LR, obtained by using Eq. 1 for arbitrary chosen value of ${\epsilon }_{m}$ and AR. In Fig. 1(a), while studying the effect of AR, ${\epsilon }_{m}$ was kept constant at 1.77. Similarly, in Fig. 1(b), the effect of ${\epsilon }_{m}$ was observed for a fixed value of AR = 2.5. It can be seen from both figures that, with the increase in the AR and ${\epsilon }_{m}$, LR shows a significant red wavelength shift.

The first step in automating the theoretical fitting to the experimentally obtained value of LR is to rearrange and thus represent the absorption coefficient seen in Eq. 1, as the product of two variables, as shown in Eq. 5. The first variable (${A}_{\lambda }^{AR}$), contains all the size and frequency dependent terms (as shown in Eq. 6), whereas the second variable (${N}^{AR}$) is the number of particles per unit volume of a given AR.

5 $$\gamma ={A}_{\lambda }^{AR}{N}^{AR},$$

where,

6 $${A}_{\lambda }^{AR}=\frac{2\pi {\epsilon }_{m}^{\frac{3}{2}}}{3\lambda }\sum _{J=A,B,C}\frac{(1/{P}_{J}^{2}){\epsilon }_{2}}{{\left({\epsilon }_{1}+\left[\frac{1-{P}_{J}}{{P}_{J}}\right]{\epsilon }_{m}\right)}^{2}+{\epsilon }_{2}^{2}}.$$

In the self-normalized absorption spectrum of a poly-disperse colloidal solution, the value of ${N}^{AR}$ varies from 0 and 1, thus indicating the contribution of GNRs of a particular AR in the total absorption. The ${N}^{AR}$ equal to 0 shows that the solution is devoid of GNRs of a given AR, whereas ${N}^{AR}$ equal to 1 indicates the mono-dispersed GNRs solution. Therefore, for a poly-dispersed colloidal solution having “$n$” varieties of ARs, the resultant absorption, at a given wavelength, say (${\lambda }_{x}$), is the cumulative effect of the contribution of the individual ARs. Mathematically, this can be represented as shown by Eq. 7:

7 $${A}_{{\lambda }_{x}}^{{AR}^{1}}{N}^{{AR}^{1}}+{A}_{{\lambda }_{x}}^{{AR}^{2}}{N}^{{AR}^{2}}+\ldots +{A}_{{\lambda }_{x}}^{{AR}^{n}}{N}^{{AR}^{n}}={A}_{{\lambda }_{x}}^{exp},$$

where, ${A}_{{\lambda }_{x}}^{{AR}^{n}}$ is the absorption of the ${n}^{th}$ AR at ${\lambda }_{x}$, assuming ${N}^{{AR}^{n}}$ is 1, i.e. the absorption of a mono-dispersed GNRs solution with respect to the ${n}^{th}$ AR GNRs. This is calculated using Eq. 6, for given ${\lambda }_{x}$ at ${\epsilon }_{m}$ = 1.77. ${N}^{{AR}^{n}}$ is the actual contribution of a ${n}^{th}$ AR, and, unknown in the present case. ${A}_{{\lambda }_{x}}^{exp}$ is the experimentally obtained value of the absorption at ${\lambda }_{x}$, obtained using an analysis of UV-Vis spectrum of a chemically-synthesized colloidal solution.

Spanning ${\lambda }_{x}$ over the entire LR wavelength range i.e. $\ge$615 nm, in $m$ steps will constitute an over-determined set of linear equations (with $n$ variables, $m$ equations; where $n\,\le \,m$). This set of over-determined equations in the matrix notation can then be expressed as:

8 $$\mathop{\underbrace{[\begin{array}{cccc}{A}_{{\lambda }_{1}}^{A{R}^{1}} & {A}_{{\lambda }_{1}}^{A{R}^{2}} & \cdots  & {A}_{{\lambda }_{1}}^{A{R}^{n}}\\ {A}_{{\lambda }_{2}}^{A{R}^{1}} & {A}_{{\lambda }_{2}}^{A{R}^{2}} & \cdots  & {A}_{{\lambda }_{2}}^{A{R}^{n}}\\ \vdots  & \vdots  & \ddots  & \vdots \\ {A}_{{\lambda }_{m}}^{A{R}^{1}} & {A}_{{\lambda }_{m}}^{A{R}^{2}} & \cdots  & {A}_{{\lambda }_{m}}^{A{R}^{n}}\end{array}]}}\limits_{{\boldsymbol{A}}}\times \mathop{\underbrace{[\begin{array}{c}{N}^{A{R}^{1}}\\ {N}^{A{R}^{2}}\\ \vdots \\ {N}^{A{R}^{n}}\end{array}]}}\limits_{{\boldsymbol{N}}}=\mathop{\underbrace{[\begin{array}{c}{A}_{{\lambda }_{1}}^{exp}\\ {A}_{{\lambda }_{2}}^{exp}\\ \vdots \\ {A}_{{\lambda }_{m}}^{exp}\end{array}]}}\limits_{{{\boldsymbol{A}}}_{exp}}$$

Equation 8, being over-determined in nature, cannot be solved to find the exact values of ${\boldsymbol{N}}$. Nevertheless, the value closest to the solution, represented by ${{\boldsymbol{N}}}^{* }$, can be found by using a Least Squares Approximation (LSA), employing Eq. 9, where (${{\boldsymbol{A}}}^{^{\prime} }$) and (${{\boldsymbol{A}}}^{-1}$) represent respectively the transpose and inverse of the matrix ${\boldsymbol{A}}$^25^.

9 $${{\boldsymbol{N}}}^{* }=({({{\boldsymbol{A}}}^{^{\prime} }{\boldsymbol{A}})}^{-1}{{\boldsymbol{A}}}^{^{\prime} }){{\boldsymbol{A}}}_{exp}.$$

However, the LSA may result in non-physical values of ${{\boldsymbol{N}}}^{* }$, such as negative values and/or those greater than unity. Therefore their optimal physical values i.e. those lying between 0 and 1, are found using Bellman’s dynamic programming Principle of Optimality (BPO)^26^.

Using BPO, whatever the value of a given element of ${\boldsymbol{N}}$, say ${N}^{{AR}^{x}}$, is chosen, the calculated values of the remaining elements of ${\boldsymbol{N}}$ will be optimal with respect to the chosen value of ${N}^{{AR}^{x}}$. This ensures that each set of values (for every ${N}^{{AR}^{x}}$) is optimized and therefore, out of all the chosen values of ${N}^{{AR}^{x}}$, the value that yields the least fitting error will be the optimal value. Mathematically, to find the optimal value of ${N}^{{AR}^{1}}$ lying between 0 and 1, it is varied between 0 to 1, using a step size of 0.001. For each step value, ${N}^{{AR}^{1}}$ is multiplied by the first row of ${\boldsymbol{A}}$ in Eq. 8 and shifted to the right-hand side, represented by ${{\boldsymbol{A}}}_{{red}}^{(1)}$ in Eq. 10. This operation modifies Eq. 8 to give Eq. 11, where ${{\boldsymbol{A}}}_{T}^{(1)}$, ${{\boldsymbol{N}}}_{T}^{(1)}$ and ${{\boldsymbol{A}}}_{{red}}^{(1)}$ are the truncated versions of ${\boldsymbol{A}}$, ${\boldsymbol{N}}$ and ${{\boldsymbol{A}}}_{exp}$ respectively.

10 $${{\boldsymbol{A}}}_{red}^{(1)}={{\boldsymbol{A}}}_{exp}-{N}^{{AR}^{1}}\left[\begin{array}{c}{A}_{{\lambda }_{1}}^{{AR}^{1}}\\ {A}_{{\lambda }_{2}}^{{AR}^{1}}\\ \vdots \\ {A}_{{\lambda }_{m}}^{{AR}^{1}}\end{array}\right],$$

11 $$\mathop{\underbrace{[\begin{array}{ccc}{A}_{{\lambda }_{1}}^{A{R}^{2}} & \cdots  & {A}_{{\lambda }_{1}}^{A{R}^{n}}\\ {A}_{{\lambda }_{2}}^{A{R}^{2}} & \cdots  & {A}_{{\lambda }_{2}}^{A{R}^{n}}\\ \vdots  & \ddots  & \vdots \\ {A}_{{\lambda }_{m}}^{A{R}^{2}} & \cdots  & {A}_{{\lambda }_{m}}^{A{R}^{n}}\end{array}]}}\limits_{{{\boldsymbol{A}}}_{{\boldsymbol{T}}}^{(1)}}\times \mathop{\underbrace{[\begin{array}{c}{N}^{A{R}^{2}}\\ \vdots \\ {N}^{A{R}^{n}}\end{array}]}}\limits_{{{\boldsymbol{N}}}_{{\boldsymbol{T}}}^{(1)}}={{\boldsymbol{A}}}_{red}^{(1)}\mathrm{.}$$

In shorthand notation, the truncated matrices of Eq. 11 can be written as,

12 $${{\boldsymbol{A}}}_{T}^{(1)}{{\boldsymbol{N}}}_{T}^{(1)}={{\boldsymbol{A}}}_{red}^{(1)}.$$

The closest unconstrained optimal values of ${{\boldsymbol{N}}}_{T}^{* }$ using the LSA, the corresponding Error (${\boldsymbol{Er}}$) and Summed Square Error (SSE) are found using the following set of equations:

13 $${{\boldsymbol{N}}}_{{\boldsymbol{T}}}^{\ast }=({(({A}_{T}^{(1)})^{\prime} {{\boldsymbol{A}}}_{{\boldsymbol{T}}}^{(1)})}^{-1}({A}_{T}^{(1)})^{\prime} ){{\boldsymbol{A}}}_{{red}}^{(1)}.$$

14 $${\boldsymbol{Er}}={{\boldsymbol{A}}}_{red}^{(1)}-{{\boldsymbol{A}}}_{T}^{(1)}{{\boldsymbol{N}}}_{T}^{* }.$$

15 $$SSE={\boldsymbol{Er}}^{\prime} {\boldsymbol{Er}}.$$

The SSE can be calculated for all the step values of ${N}^{{AR}^{1}}$ and among them, the one which gives the least SSE is taken as the optimized value of ${N}^{{AR}^{1}}$, represented by ${N}_{opti}^{{AR}^{1}}$. Since, in calculating ${N}_{opti}^{{AR}^{1}}$, all the unconstrained possibilities of ${N}^{{AR}^{2}}$ to ${N}^{{AR}^{n}}$ are considered, there is no need to recalculate the value of ${N}^{{AR}^{1}}$ while calculating their optimal values. For example, to calculate the optimal value of ${N}^{{AR}^{2}}$, it is varied between 0 to 1 with a step size of 0.001. Since, ${N}_{opti}^{{AR}^{1}}$ has already been calculated in the previous step, for each step value of ${N}^{{AR}^{2}}$, the first row of A (in Eq. 8) is multiplied by ${N}_{opti}^{{AR}^{1}}$, the second row is multiplied by ${N}^{{AR}^{2}}$ and shifted to the right-hand side, i.e. subtracted from ${{\boldsymbol{A}}}_{exp}$, as shown in Eq. 16. This operation will reduce the matrices ${\boldsymbol{A}}$, ${\boldsymbol{N}}$, ${{\boldsymbol{A}}}_{exp}$ in Eq. 8 to ${{\boldsymbol{A}}}_{T}^{(2)}$, ${{\boldsymbol{N}}}_{T}^{(2)}$, ${{\boldsymbol{A}}}_{red}^{(2)}$ respectively, as shown in Eq. 17.

16 $${{\boldsymbol{A}}}_{red}^{(2)}={{\boldsymbol{A}}}_{exp}-{N}_{opti}^{{AR}^{1}}\left[\begin{array}{c}{A}_{{\lambda }_{1}}^{{AR}^{1}}\\ {A}_{{\lambda }_{2}}^{{AR}^{1}}\\ \vdots \\ {A}_{{\lambda }_{m}}^{{AR}^{1}}\end{array}\right]-{N}^{{AR}^{2}}\left[\begin{array}{c}{A}_{{\lambda }_{1}}^{{AR}^{2}}\\ {A}_{{\lambda }_{2}}^{{AR}^{2}}\\ \vdots \\ {A}_{{\lambda }_{m}}^{{AR}^{2}}\end{array}\right]$$

17 $$\mathop{\underbrace{[\begin{array}{ccc}{A}_{{\lambda }_{1}}^{A{R}^{3}} & \cdots  & {A}_{{\lambda }_{1}}^{A{R}^{n}}\\ {A}_{{\lambda }_{2}}^{A{R}^{3}} & \cdots  & {A}_{{\lambda }_{2}}^{A{R}^{n}}\\ \vdots  & \ddots  & \vdots \\ {A}_{{\lambda }_{m}}^{A{R}^{3}} & \cdots  & {A}_{{\lambda }_{m}}^{A{R}^{n}}\end{array}]}}\limits_{{{\boldsymbol{A}}}_{{\boldsymbol{T}}}^{(2)}}\times \mathop{\underbrace{[\begin{array}{c}{N}^{A{R}^{3}}\\ \vdots \\ {N}^{A{R}^{n}}\end{array}]}}\limits_{{{\boldsymbol{N}}}_{{\boldsymbol{T}}}^{(2)}}={{\boldsymbol{A}}}_{red}^{(2)}$$

The unconstrained value of ${{\boldsymbol{N}}}_{T}^{(2)}$ using the LSA, ${\boldsymbol{Er}}$ and the SSE corresponding to each step is found following the steps taken and shown in Eqs 13–15 and the resultant value that then gives a minimum value of the SSE is selected as the optimal value of ${N}^{{AR}^{2}}$. Similarly, in calculating ${N}^{{AR}^{3}}$, ${N}^{{AR}^{1}}$ and ${N}^{{AR}^{2}}$ are kept equal to the optimal values found from the previous step. Likewise, after calculating all the values of ${\boldsymbol{N}}$, i.e. ${N}^{{AR}^{1}}$ to ${N}^{{AR}^{n}}$, the final resultant (fitted) curve is obtained by post multiplying it by ${\boldsymbol{A}}$, as per Eq. 8.

In order to determine the effect of the solvent on the process, the fitted curve for ${\epsilon }_{m}$ taking values from 1.77 to 2.3 with a step size of 0.05 is generated following the above procedure. The error and SSE values determined from the experimental and the fitted curve (${\boldsymbol{Er}}$ = ${{\boldsymbol{A}}}_{exp}\ -\ {{\boldsymbol{A}}}_{fitted}$; SSE = ${\boldsymbol{Er}}^{\prime} {\boldsymbol{Er}}$) are calculated for all the step values of ${\epsilon }_{m}$ and among them, the value of ${\epsilon }_{m}$ giving the minimum SSE is selected to generate the final fitted curve. The value of ${\boldsymbol{N}}$ corresponding to the final fitted curve is taken as the contribution of the individual ARs.

The algorithm was coded in MATLAB^27^ and the source-code is provided in the Supplementary Material.

## Results and Discussions

### Comparison with TEM measurement

**Figure 2:**
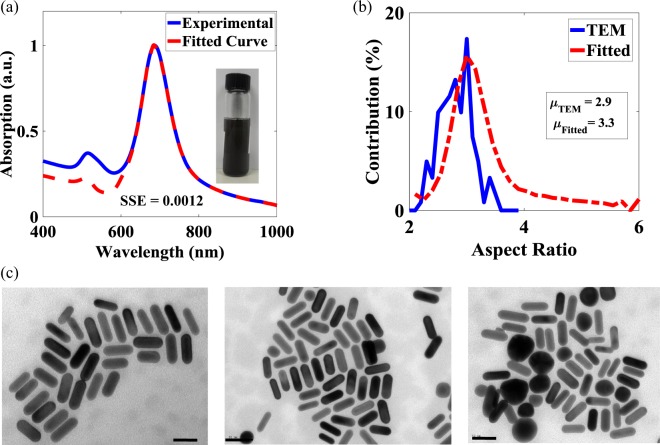
(**a**) The UV-Vis absorption spectrum of the chemically synthesized sample (solid line) and the spectrum obtained by fitting the longitudinal resonance (dash line). Inset is shown a photograph of the GNRs solution (**b**) AR distributions curve obtained through the measurement with the TEM images measurement (solid line) and fitting of The UV-Vis spectrum (dashed line). The legend shows the mean value for both graphs. (**c**) TEM images taken at three different places on the TEM grid. The scale bar represents 50 nm.

**Figure 3:**
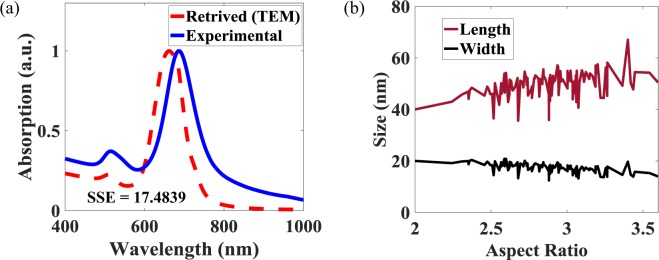
(**a**) The UV-Vis absorption spectrum of the chemically synthesized sample (solid line) compared with spectrum retrieved from AR distribution obtained from TEM measurement (dashed line). (**b**) Dependence of length and width of GNRs on AR obtained from TEM measurement.

The experimental validation of the algorithm developed was performed by comparing the AR distribution obtained from the use of the algorithm with that obtained by by manually measuring 121 GNRs from TEM images captured at three different places on the TEM grid. Figure 2(a) shows an experimentally-obtained UV-Vis spectrum and the absorption spectrum obtained by fitting an experimental data points in the LR region. As it can be seen from the figure, both graphs match well (SSE = 0.0012) in the LR region. Further, a picture of the actual synthesized colloidal solution is shown in the inset. The black color of the solution indicates the presence of a low number of GNPs. Figure 2(b) shows the distribution of the ARs obtained by measuring GNRs from TEM images and those obtained from the use of algorithm whereas, Fig. 2(c) shows the corresponding TEM images used for the ARs measurements. It can be seen in Fig. 2(b) that, the algorithm used correctly predicted the most probable AR, the value of which is 3. However, the overall AR distribution obtained from the TEM images is shifted towards lower ARs and consequently, the mean value of the distribution obtained by the use of TEM is lower than that of the obtained from developed algorithm (${\mu }_{TEM}$ = 2.9; ${\mu }_{Fitted}$ = 3.3). This mismatch is due to the inability of the TEM to examine the macroscopic volume in comparison to using longitudinal plasmon resonance of UV-Vis spectrum, which is very much sensitive to the macroscopic ARs distribution seen in the colloidal solution. To corroborate the above claim, the UV-Vis spectrum was reconstructed by substituting the contribution obtained from TEM measurement in Eq. 8, matching it with the experimentally-obtained UV-Vis spectrum. As shown in Fig. 3(a), UV-Vis spectrum retrieved using the TEM measurements deviates significantly from the experimental UV-Vis spectrum ($SS{E}_{TEM}$ = 17.4839 is much larger than the value obtained from the algorithm $SS{E}_{algorithm}$ = 0.0012). The inability to obtain a statistically accurate AR distribution from the TEM measurement was also reported by Eustis & Sayed^22^ for 5 different samples, showing longitudinal resonance around 630 nm, 700 nm, 850 nm, 900 nm & 1000 nm. In this work, these UV-Vis spectra have been reconstructed after extracting data using a Webplot digitizer^28^, followed by the use of the algorithm developed to calculate the AR distribution. (As shown in the Supplementary Fig. 1, there is good agreement between the AR distributions obtained by Eustis & Sayed^22^ and that of this work. This further validate the reliability of the developed algorithm).

It is important to note that due to the growth kinematics of seed-based synthesis, an AR cannot have a limitless combination of length and width, because of the same average volume of GNRs for a given colloidal solution^4,22^. Therefore, any increase in the AR is caused by an increase in length and a correlated decrease in the width, as can be seen from the TEM measurements of the GNRs described in Fig. 3(b).

### Comparison with DLS measurement

**Figure 4:**
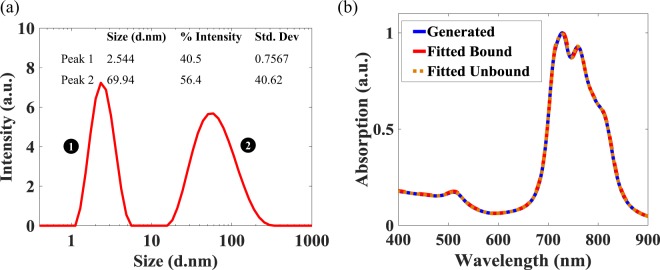
(**a**) Size Distribution of chemically synthesized GNRs obtained by using the DLS technique (**b**) Quality of the fitting of the Absorption spectra generated by the test program, obtained by fitting the generated curve with the bounded and the unbounded coefficients.

The size distribution of the chemically synthesized sample obtained from DLS measurement is shown in Fig. 4(a). It can be seen from the graph that the DLS measurement reports two peaks centered around 2.544 nm and 69.94 nm. The first peak can be attributed to seed GNPs which could not grow due to insufficient amount of gold in the growth solution, whereas second peak accounts for fully developed GNRs and GNPs. Since, DLS gives the size distribution with respect to the hydrodynamic radius, i.e. the radius of an equivalent sphere having same diffusion coefficient, it is difficult to obtained the AR distribution from the graph shown, especially when spherical and cubic gold impurities are present in the colloidal solution. For example, few spherical GNPs impurities can be seen in right-hand TEM image, shown in Fig. 2(c). The inability of DLS to distinguish shape makes it unsuitable to measure the AR distribution. In contrast to the DLS measurement, the algorithm developed here is unaffected by the presence of large spherical GNPs impurities, because they do not exhibit a LR.

The algorithm developed was further benchmarked with the results of a numerical technique, as described in the next subsection, to circumvent the limitations discussed above and associated with the TEM and DLS measurements.

### Theoretical bench-marking of the Algorithm

For the effective theoretical bench-marking of the algorithm developed, the absorption spectrum, resembling that of a chemically synthesized poly-dispersed colloidal solution, was generated numerically from the combination of 11 GNRs samples having AR values ranging between 3 and 4 (with a regular interval of 0.1), using Eq. 8. In Eq. 8, ${\boldsymbol{N}}$ was randomly generated by the use of an in-built random function generator on MATLAB^27^ and ${\boldsymbol{A}}$ was generated from Eq. 6 over the wavelength range from 400 to 900 nm, with a step size of 1 nm, at ${\epsilon }_{m}$ = 2.1.

**Figure 5:**
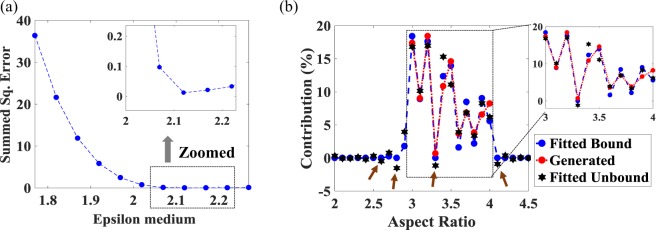
(**a**) Fitted SSE as function of ${\epsilon }_{m}$ for the bounded coefficients. Inset shows the obtained minimum SSE for ${\epsilon }_{m}$ = 2.12. (**b**) Left: the figure compares the generated (test) AR contribution with that generated by the algorithm for the bounded and unbounded coefficients. Right: ‘Zooming in’ on the 3 $\le$ AR $\le$ 4 region from the main (Left) figure.

The spectrum generated was input to the algorithm developed to retrieve the value of ${\epsilon }_{m}$ and the corresponding (bounded) values of ${\boldsymbol{N}}$. Further, to validate the point mentioned in the previous section i.e. solving the over-determined set of linear equations without any constrain may result in non-physical values, the LSA technique was used to obtained the unbounded values of ${\boldsymbol{N}}$. Both the unbounded and bounded values of ${\boldsymbol{N}}$ were cross-compared with the known input (randomly generated) values of ${\boldsymbol{N}}$ to determine the accuracy of the methods used. Figure 4(b) shows the generated and retrieved absorption spectra. It can be seen from this that all three spectra i.e. the generated spectrum and the spectra obtained using the developed algorithm and LSA match very closely at ${\epsilon }_{m}$ = 2.12. The value of ${\epsilon }_{m}$ = 2.12 was obtained by analyzing the variation of the values of the SSE with respect to ${\epsilon }_{m}$, as shown in Fig. 5(a). The minimum SSE value was obtained at ${\epsilon }_{m}$ = 2.12 (and this is shown in the inset). It matches very well with the value of ${\epsilon }_{m}$ = 2.1 used for generating the input spectrum.

Figure 5(b) shows the input and retrieved values (i.e the contribution), of ${\boldsymbol{N}}$, represented as a percentage, for different values of AR. The blue solid spheres shows the value of the contribution obtained from the developed (bounded) algorithm. It can be seen from the figure that, outside the input regime i.e. AR $<$ 3 & AR $>$ 4, the developed algorithm correctly predicted the absence of any GNRs (i.e. a zero contribution) whereas for 3 $\le$ AR $\le$ 4, the values obtained from the algorithm are in good agreement with the input values (represented by the red solid spheres), as shown in zoomed right-hand figure and from Table 1. However, if the contributions obtained from the LSE is considered (shown by black stars in the main and the zoomed figure), it gives a negatives contribution for some values of the AR, as indicated by brown arrows in Fig. 5(b). Moreover, as given in Table 1, the SSE obtained from the Fitted Bound is much lower than the SSE obtained from Fitted Unbound situation ($SS{E}_{bound}$ = 1.833; $SS{E}_{unbound}$ = 7.59).This result shows that the algorithm which has been developed is capable of correctly retrieving the contribution from the UV-Vis absorption spectrum of the poly-dispersed colloidal solution.

**Table 1:** AR and corresponding contribution obtained from Generated, Fitted Bound, & Fitted Unbound techniques

AR	Generated	Fitted Bound	Error	Fitted Unbound	Error
3	17.17	18.39	0.059	16.8	$-0.033$
3.1	9.008	8.9	$-0.01$	10.14	0.1257
3.2	18.41	17.6	$-0.04$	16.98	$-0.078$
3.3	0.6756	0	$-1$	$-1.137$	$-2.683$
3.4	10.84	12.34	0.138	15.3	0.4114
3.5	14.59	13.92	$-0.05$	11.11	$-0.239$
3.6	3.651	1.582	$-0.57$	3.916	0.0726
3.7	6.852	8.465	0.235	6.849	$-4.00E \mbox{-} 04$
3.8	3.822	2.176	$-0.43$	3.375	$-0.117$
3.9	6.555	9.019	0.376	8.284	0.2638
4	8.223	5.578	$-0.32$	6.184	$-0.248$
		**SSE**	1.833		7.59

### Application on additional synthesized samples

**Figure 6:**
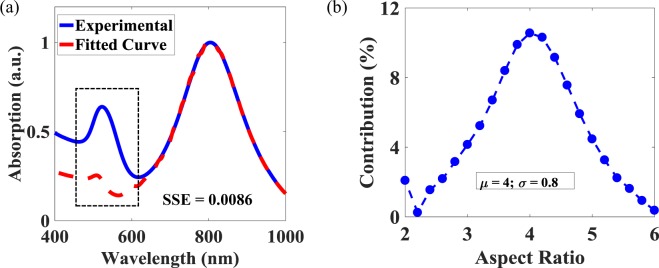
(**a**) The absorption spectra of chemically-synthesized sample (solid line), juxtaposed with the spectra obtained by fitting the longitudinal resonance (dashed line). The dotted section highlights the mismatch around transverse resonance. (**b**) AR distributions curve. The legend shows mean value and standard deviation. For both graphs, ${\epsilon }_{m}=1.92$ was used.

After benchmarking, the bounded technique was used to determine the AR distributions in two further chemically-synthesized samples. Figure 6(a) shows the LR fitting used in the case of a sample having well-separated LR and TR peaks. As can be seen from the figure, in the LR regime i.e. where the wavelength is $\ge$615 nm, the fitted curve matches closely the experimentally-obtained absorption spectrum (SSE = 0.0086). Since, only the LR is used to obtain the contribution (as a percentage) of the individual ARs, extending the fitted spectrum to the TR spectral region (which is less than the wavelength of 615 nm) will give the ideal spectrum, i.e. the spectrum obtained in the absence of the GNPs (1 $\le$ AR $<$ 2). Therefore, the mismatch in the TR regime between the fitted and the experimental curves can be used to visualize quantitatively the magnitude of the GNPs present in the colloidal solution, as shown as the dotted region in Fig. 6(a). This can be described as, in the absence of any GNPs, the experimental and the fitted curves would be matched closely around the TR as well. From the mismatch at the TR in Fig. 6(a), it can be concluded that a significant number of GNPs are present in the solution. Figure 6(b) shows the contribution (again as a percentage) of the AR present, where the calculated mean ($\mu$) AR of the solution is 4, with a standard deviation ($\sigma$) of 0.8.

**Figure 7:**
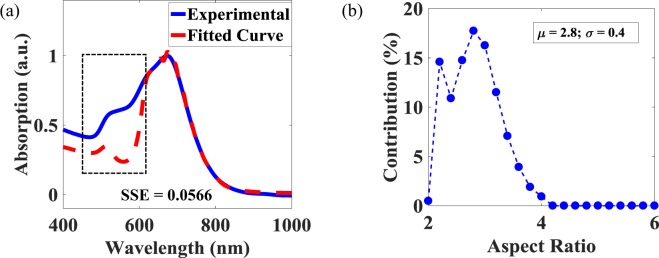
(**a**) Measured (solid line) and fitted (dashed line) absorption spectra, for a low AR sample. Dotted section qualitatively highlights the amount of particles having AR $\le$ 2. (**b**) Contribution of various AR values to obtain the fitted curve in (**a**). The SSE was a minimum for ${\epsilon }_{m}=1.97$.

Figure 7(a) shows the quality of the curve fitting in the case of a solution where the TR and the LR cannot readily be distinguished. It can be seen that for a wavelength $\ge$615 nm, the theoretical curve fits well to the experimentally obtained spectrum. The mismatch between the fitted and the experimental curves around the TR, as illustrated by the dotted section on the graph, shows the presence of the large amount of GNPs. Figure 7(b) shows the AR contributions and, as expected, by comparison to an analysis of the previous colloidal solution, this solution has an abundance of less elongated GNRs ($\mu$ = 2.8; $\sigma$ = 0.4) and the amount of GNRs seen with an AR value greater than 4.2 is almost zero.

## Conclusion

The research carried out has shown that the algorithm developed in this paper using Bellman’s dynamic technique is an effective approach for the rapid determination of the AR distribution of a synthesized GNRs colloidal solution by theoretically fitting the LR regime of the absorption spectrum. The developed algorithm, after numerical and experimental benchmarking, was applied additionally to two in-house synthesized solutions to determine the mean and standard deviations of the ARs. This technique has been shown to give both a cost-effective and rapid way to determine the quality of the synthesized solution as it avoids the need for more expensive TEM analysis of all of the samples synthesized .

## Methods

### Chemical synthesis

GNRs were synthesized by using the seed-mediated method reported by Jie Cao *et al*.^29^. In brief, the seed solution was prepared by reducing a solution containing 5 mL of 0.2 M Cetyltrimethylammonium Bromide (CTAB) and 5 mL of 0.5 mM $HAuC{l}_{4}\cdot 3{H}_{2}O$, with 600 $\mu$L of 0.01 M ice-cold $NaB{H}_{4}$. After stirring the solution for an additional 2 minutes at 900 rpm, the seed solution was left undisturbed for 3 hours.

The growth solution was prepared by mixing a known amount of 20 mM $AgN{O}_{3}$ solution, 30 mL of 0.2 M CTAB solution and 30 mL of 1 mM $HAuC{l}_{4}\cdot 3{H}_{2}O$ in the same sequence. To the mixed solution, 420 $\mu$L of 0.0788 M ascorbic acid solution was added, while stirring at 350 rpm. This step will change the colour of solution from bright yellow to colorless. Further, 100 $\mu$L of seed solution was added to the growth solution and the resulting solution was left undisturbed for 12 hours. Since CTAB is insoluble in deionized (DI) water at room temperature, all the synthesis steps were performed in a water bath maintained at a constant temperature of $2{8}^{\circ }{\rm{C}}$.

The excess of CTAB present in the GNR solution was removed by using two rounds of centrifugation at 3700g for 20 min for each round. After each round, the supernatant was decanted and the GNRs deposited at the bottom of the centrifuge tube were re-dispersed in the same quantity of DI water.

### Characterization

The absorption spectra of the GNRs solutions were measured by using a LAMBDA 35 UV-Vis spectrometer (Perkin Elmer Inc.) monitoring over the wavelength range from 400 nm to 1000 nm, in steps of 1 nm. The TEM images were taken from JEOL at accelerating voltages of 80kV and yielding 400k magnification. The dimensions of the GNRs from the TEM images were measure using ImageJ, which is an open source image processing software inspired by NIH image^30^. The DLS measurement was performed using a ZetaSizer Nano ZEN 3600 (Malvern Instrument).

## Supplementary information

Supplementary Information

## Data Availability

Data will be provided on reasonable request. MATLAB source-code is given in the Supplementary Information.
