# Supplementary material for: Determination of the Aspect-ratio Distribution of Gold Nanorods in a Colloidal Solution using UV-visible absorption spectroscopy
Source: Sci Rep. 2019 Nov 25;9:17469. doi: 10.1038/s41598-019-53621-4 (PMC6877556; doi:10.1038/s41598-019-53621-4)
Supplement: Supplementary file 1 — Supplementary Information [file 41598_2019_53621_MOESM1_ESM.pdf]

Determination of the Aspect-ratio Distribution of Gold Nanorods in a Colloidal Solution using UV-visible absorption spectroscopy

Rahul Kumar[1,*], Leonardo Binetti[2], Hien T Nguyen[1], Lourdes S M Alwis[2], Arti Agrawal[3], Tong Sun[1], and Kenneth T V Grattan[1]

[1]School of Mathematics, Computer Science & Engineering, City, University of London, London, EC1V 0HB, United Kingdom
[2]Edinburg Napier University, School of Engineering and the Built Environment, Edinburgh, EH11 4BN, Edinburgh, United Kingdom
[3]Department of Engineering and Information Technology, University of Technology Sydney, 15 Broadway, Ultimo NSW 2007, Australia

[*]Rahul.Kumar@city.ac.uk

Supplementary Material:

Figure:

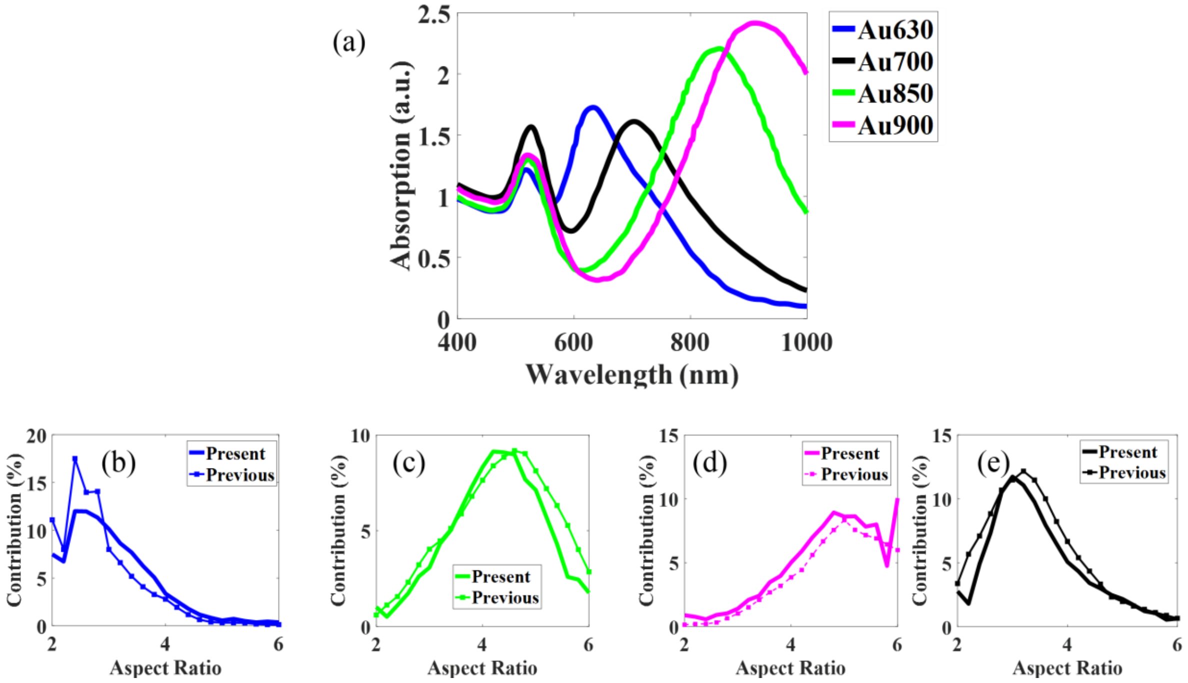

Figure 1: (a) The UV-visible spectra extracted from the work of Eustin & Sayed[1]. (b)-(e) Comparison of AR distribution obtained from the algorithm developed in present work with the one reported previously in reference [1].

Source-Code (written in MATLAB[2]):

```
close all; clc;clearvars;
```

```
%%%% Reading exp. value of RI from Johnson & Christy (1972) given at the end of program
```

```
experimental_refractive_index = data_john_christy();
wavelength_john = experimental_refractive_index (:,1);
n_exp = experimental_refractive_index (:,2);
k_exp = experimental_refractive_index (:,3);

%%%% reading experimental UV-Vis values from external file
filename = 'example.Sample'; %%% UV_VIS spectrum file name (without header):
filename = [filename '.csv']; %%%%  UV-Vis file format (wavelength, Absorption (nm))
UV_Vis_data_exp = load (filename);

% finding row number corresponding to wavelength 400 nm, 617 nm & 1000
...wavelength_index is a subroutine, whose body is defined in the end

index_400 = wavelength_index(UV_Vis_data_exp (:,1),400);
index_618 = wavelength_index(UV_Vis_data_exp (:,1),618);
index_1000 = wavelength_index(UV_Vis_data_exp (:,1),1000);

%extracting data from UV-VIS spectum file
%LR stands for Longitudinal Resonance

absorb_exp_LR = UV_Vis_data_exp (index_1000:index_618,2);
absorb_exp1_normalized = absorb_exp_LR./max(absorb_exp_LR);
absorb_exp_complete_spectrum_normalized = UV_Vis_data_exp
(index_1000:index_400,2)./max(UV_Vis_data_exp (index_1000:index_400,2));
lambda_complete = UV_Vis_data_exp (index_1000:index_400,1); %% lambda = 400 nm to 1000 nm
lambda_LR = UV_Vis_data_exp (index_1000:index_618,1);
len_lamba = length(lambda_LR);

%interpolation of experimental refractive index data over 300 to 900 at step of 1
ref_real_interpolated = interp1(wavelength_john, n_exp,lambda_LR,'spline');
ref_imag_interpolated = interp1(wavelength_john, k_exp,lambda_LR,'spline');

%calculating relative permittivity from interpolated refractive index data
epsilon_exp_complete = (ref_real_interpolated+1i.*ref_imag_interpolated).^2;
epsilon_real_interpolated = real(epsilon_exp_complete);
epsilon_imag_interpolated = imag(epsilon_exp_complete);

%%%%%%%%%%%%%%%% Applying Gans theory  %%%%%%%%%%%%%%
AR = 2 : 0.2 : 6;
epsilon_medium = 1.77:0.05:2.1;
Absob_coff_transpose = zeros(length(AR),length(lambda_LR));
Absob_coff_transpose_normalized = zeros(length(AR),length(lambda_LR)); %% Normalized
```

```
Absorption Coefficient
N = 1; % Initial contribution
V = 1; %volume
% Loop to find AR and Epsilon_medium having minimum Summed Square Error %%%%
% Refer main paper for algorithm
for k = 1:length(epsilon_medium)
    for i = 1:length(AR)
        elong = sqrt (1-(1/(AR(i)^2)));
        PA = ((1-elong^2)/elong^2)*(1./(2*elong)* log ((1+elong)/(1-elong))-1);
        PB = (1-PA)/2;
        PC = PB;
        coff_A = ((1/PA^2).*epsilon_imag_interpolated)./((epsilon_real_interpolated + ((1-
PA)/PA)*epsilon_medium(k)).^2 + epsilon_imag_interpolated.^2);
        coff_B = ((1/PB^2).*epsilon_imag_interpolated)./((epsilon_real_interpolated + ((1-
PB)/PB)*epsilon_medium(k)).^2 + epsilon_imag_interpolated.^2);
        coff_C = coff_B;
        Absob_coff_transpose (i,:) = (2*pi*N*V*(epsilon_medium(k))^(3/2))./(3.*lambda_LR)
.*(coff_A + coff_B + coff_C);
        Absob_coff_transpose_normalized (i,:) = Absob_coff_transpose (i,:) ./
max(Absob_coff_transpose (i,:));
    end
    Absob_coff_norm = transpose(Absob_coff_transpose_normalized);
    factor = 0:0.001:1;
    absorb_exp1_norm_new = absorb_exp1_normalized;
    for i = 1:(length(AR))
        if (i<length(AR))
            trun_coff = Absob_coff_norm(:,i+1:length(AR));
        else
            trun_coff = Absob_coff_norm(:,length(AR));
        end
        trun_coff_tran = transpose (trun_coff);
        factor_1 = factor .* Absob_coff_norm(:,i);

        for j = 1:length(factor)
            absorb_exp1_norm_new_1 = absorb_exp1_norm_new-factor_1(:,j);
            if (i<length(AR))
                P1 =(trun_coff_tran*trun_coff)\trun_coff_tran;
                error_value_1 = P1*absorb_exp1_norm_new_1;
                error_value = trun_coff*error_value_1;
            else
                absorb_exp1_norm_new_1 = absorb_exp1_norm_new;
                error_value = factor_1(:,j);
            end
            SSE(i,j) = transpose(absorb_exp1_norm_new_1-error_value)*(absorb_exp1_norm_new_1-
error_value);
        end
        [min_SSE,min_index] = min(SSE(i,:));
        freq(k,i,1)= factor(min_index);
        absorb_exp1_norm_new = absorb_exp1_norm_new-factor_1(:,min_index);
    end
    fitted_curve= Absob_coff_norm * transpose(freq(k,:));
    error_final = absorb_exp1_normalized-fitted_curve;
    SSE_final(k) = transpose(error_final)*(error_final);
end
[min_SSE_eps,min_index_eps] = min(SSE_final);
epsilon_minimum = epsilon_medium(min_index_eps);
```

```
%%%% for plotting complete curve i.e lambda = 400 nm to 1000 nm

%interpolation of experimental epsilon data over 300 to 900 at step of 1
ref_real_interpolated = interp1(wavelength_john, n_exp,lambda_complete,'spline');
ref_imag_interpolated = interp1(wavelength_john, k_exp,lambda_complete,'spline');

%calculating relative permittivity from ref. index data
epsilon_exp_complete = (ref_real_interpolated+1i.*ref_imag_interpolated).^2;
epsilon_real_interpolated = real(epsilon_exp_complete);
epsilon_imag_interpolated = imag(epsilon_exp_complete);

% intialization corresponding to complete lambda
Absob_coff_transpose = zeros(length(AR),length(lambda_complete));
Absob_coff_transpose_normalized = zeros(length(AR),length(lambda_complete));

% Calculating complete UV-Vis form fitted contribution

for i = 1:length(AR)
    elong = sqrt (1-(1/(AR(i)^2)));
    PA = ((1-elong^2)/elong^2)*(1./(2*elong)* log ((1+elong)/(1-elong))-1);
    PB = (1-PA)/2;
    PC = PB;
    coff_A = ((1/PA^2).*epsilon_imag_interpolated)./((epsilon_real_interpolated + ((1-
PA)/PA)*epsilon_medium(min_index_eps)).^2 + epsilon_imag_interpolated.^2);
    coff_B = ((1/PB^2).*epsilon_imag_interpolated)./((epsilon_real_interpolated + ((1-
PB)/PB)*epsilon_medium(min_index_eps)).^2 + epsilon_imag_interpolated.^2);
    coff_C = coff_B;
    Absob_coff_transpose (i,:) =
(2*pi*N*V*(epsilon_medium(min_index_eps))^(3/2))./(3.*lambda_complete) .*(coff_A + coff_B +
coff_C);
    Absob_coff_transpose_normalized (i,:) = Absob_coff_transpose (i,:) ./
max(Absob_coff_transpose (i,:));
end

Absob_coff_norm = transpose(Absob_coff_transpose_normalized);
fitted_curve_1= Absob_coff_norm * transpose(freq(min_index_eps,:));

% calculating mean & standard deviation
mean = round(sum(AR.*freq(min_index_eps,:))/sum(freq(min_index_eps,:)),1);
variance_sum = (freq(min_index_eps,:).*(AR-mean).^2);
std_deviation = round(sqrt(sum(variance_sum)/sum(freq(min_index_eps,:))),1);


%%%%% plotting figures %%%%%%%%%%%%%

% plotting uv-vis spectrum
figure ();
p = plot (lambda_complete,absorb_exp_complete_spectrum_normalized,lambda_complete,
fitted_curve_1,'Linewidth',6);
p(1).LineStyle = '-';
p(1).LineWidth = 6;
p(1).Color ='[0,0,1]';
p(2).LineStyle = '--';
p(2).LineWidth = 6;
p(2).Color ='[1,0,0]';
set ( gca , 'FontSize' , 30 , 'fontweight' , 'b' , 'FontName' , 'Times New Roman') ;
```

```
xlabel ( 'Wavelength (nm)' , 'FontSize' , 30 , 'fontweight' , 'b' ,'FontName' , 'Times New
Roman') ;
ylabel ( 'Absorption (a.u.)', 'FontSize' , 30 , 'fontweight' , 'b', 'FontName' , 'Times New
Roman' ) ;
legend ( 'Experimental' ,'Fitted Curve') ;
xlim([400,1000]);
dimension = [0.2 0.5 0.3 0.3];
str = {'SSE:', num2str(min_SSE_eps)};
annotation('textbox',dimension,'String',str,'FitBoxToText','on','FontSize' , 20 ,
'fontweight' , 'b' ,'FontName' , 'Times New Roman');


% plotting contribution
figure();
p = plot (AR,(freq(min_index_eps,:)./sum(freq(min_index_eps,:)))*100);
p(1).LineStyle = '--';
p(1).LineWidth = 2;
p(1).Color ='[0,0,1]';
p(1).Marker = 'o';
p(1).MarkerSize = 10;
p(1).MarkerEdgeColor = '[0,0,1]';
p(1).MarkerFaceColor = '[0,0,1]';
set ( gca , 'FontSize' , 30 , 'fontweight' , 'b' , 'FontName' , 'Times New Roman') ;
xlabel ( 'Aspect Ratio' , 'FontSize' , 30 , 'fontweight' , 'b' ,'FontName' , 'Times New
Roman') ;
ylabel ( 'Contribution (%)', 'FontSize' , 30 , 'fontweight' , 'b', 'FontName' , 'Times New
Roman' ) ;
dimension = [0.2 0.5 0.3 0.3];
str = {'\mu / \sigma:', num2str(mean) num2str(std_deviation)};
annotation('textbox',dimension,'String',str,'FitBoxToText','on','FontSize' , 20 ,
'fontweight' , 'b' ,'FontName' , 'Times New Roman');




%%%% GOld refractive index were taken from following reference
% P.B. Johnson and R.W. Christy, "Optical constants of the noble metals," Phys. Rev. B, vol.
6, pp. 4370, 1972.
% Data format [wavelength (nm), real (refractive index), img (refractive_index)]

function y = data_john_christy()
y = [983.968254       0.22    6.350
    891.942446 0.17    5.663
    821.0596026       0.16    5.083
    755.9756098       0.14    4.542
    704.4318182       0.13    4.103
    659.4680851       0.14    3.697
    616.8159204       0.21    3.272
    582.0657277       0.29    2.863
    548.5840708       0.43    2.455
    520.9243697       0.62    2.081
    495.9200000 1.04   1.833
    471.4068441       1.31    1.849
    450.8363636       1.38    1.914
    430.4861111       1.45    1.948
    413.2666667       1.46    1.958
    397.3717949       1.47    1.952
```

```
    381.4769231        1.46    1.933
    367.8931751        1.48    1.895
    354.2285714        1.5     1.866
    342.4861878        1.48    1.871
    331.4973262        1.48    1.883
    320.3617571        1.54    1.898
    310.726817 1.53    1.893
    300.9223301        1.53    1.889
    292.4056604        1.49    1.878];
end

% wavelength subroutine Body
function y = wavelength_index(wavelen, wave)
[~, index_400] = min (abs(wavelen(:,1)-wave));
y = index_400;
end
```

Reference:

[1] Eustis, S. & El-Sayed, M. A. Determination of the aspect ratio statistical distribution of gold nanorods in solution from a theoretical fit of the observed inhomogeneously broadened longitudinal plasmon resonance absorption spectrum. *J. Appl. Phys.* **100**, 044324 (2006).

[2] The Mathworks, Inc., Natick, Massachusetts. MATLAB *version 9.1.0.441655 (R2016b)* (2016).
